# Previously introduced braconid parasitoids target recent olive fruit fly (*Bactrocera oleae*) invaders in Hawai’i

**DOI:** 10.1038/s41598-023-49999-x

**Published:** 2023-12-18

**Authors:** Dara G. Stockton, Charlotte Aldebron, Rosemary Gutierrez-Coarite, Nicholas C. Manoukis

**Affiliations:** 1 Tropical Crop and Commodity Protection Research Unit, Daniel K. Inouye Pacific Basin Agricultural Research Center, USDA-ARS, 64 Nowelo St., Hilo, HI 96720 USA; 2 Department of Tropical Agriculture and Soil Science, University of Hawai’i at Manoa, Kahalui Extension Office, 310 Kaahumanu Ave. Bldg. 214, Kahului, HI 96732 USA

**Keywords:** Agroecology, Tropical ecology

## Abstract

The olive fruit fly *Bactrocera oleae* (Diptera: Tephritidae) was detected on Maui and Hawai’i Islands in 2019, affecting yields and quality of the state’s emerging olive oil industry. Given previous parasitoid releases to control other invasive frugivorous tephritids in Hawai’i, we were interested in determining whether these parasitoids were naturally targeting recent olive fly invaders in field, if local olive cultivar differences affected parasitization rates, and if there was a seasonal pattern of parasitization that could inform future management decisions. To address these questions, we collected data from olive growing in Hawai’i during 2021 and 2022. During the fruiting season we collected monthly samples and reared out *B. oleae* in the lab. We detected two previously introduced braconid wasps: first *Diachasmimorpha tryoni* during 2021 and 2022 and later *Fopius arisanus* during the 2022 collection. Cultivar effects were limited to a single site in our study, where more *D. tryoni* were reared from ‘Arbequina’ olives. Seasonality of olive fruit fly and parasitoid activity was earlier in lower elevation sites, as expected based on tree phenology and temperature-dependent insect development. This represents the first report of *D. tryoni* parasitism activity against *B. oleae* and may reflect elevational effects combined with the ecological complexity in interactions between multiple invasive arthropod pests, their invasive and cultivated plant hosts, and introduced braconid parasitoids.

## Introduction

The olive fruit fly, *Bactrocera oleae* Rossi (Diptera: Tephritidae), is an invasive member of the tribe Dacini, which although native to Africa has long been naturalized in Southern Europe^1,2^ It was introduced to the Americas through California during the late 1990s^3–5^. In Hawai’i, *B. oleae* was first detected in August 2019 at the Lālāmilo Research Station in Waimea on the big island of Hawaiʻi and then in October of the same year on the island of Maui^6^. Since then, *B. oleae* have become widespread in olive groves on both islands where they appear to have become established^7^. Prior to the introduction of *B. oleae*, there were few pests or diseases of olives in Hawai’i, making it a good prospective crop for diversified agriculture on the islands^8^, especially because of the nature of agricultural economics in the islands, where sustainable, low-cost management options are ideal.

*Bactrocera oleae* is considered a monophagous, specialist species, as it relies solely on the fruit of trees in the genus *Olea* for larval development^9^. Sexually mature females use their specialized ovipositor to “sting” ripening olives and lay eggs directly under the surface of the skin^10^. The larvae then consume the fruit as they develop, leading to substantial yield and quality losses, both from direct feeding damage and indirectly due to the introduction of bacteria and fungal pathogens that further degrade the fruit^11–14^. On average, *B. oleae* complete their development in within 2–3 weeks and has four or more generations per year^15^. While present year-round in temperate climates, adults are most abundant during September–November, coinciding with maximum fruit availability^16^. In California, and presumably now Hawai’i, olive fruit fly is considered the most significant pest of olives^17^.

Due to its cosmopolitan nature and long-standing presence and impact as a pest in Africa, Europe and Asia^18^, *B. oleae* is a well-studied species and multiple management strategies have been developed^9,16^ including various chemical insecticides^19–21^, baits/lures for mass trapping by growers^22,23^, and the implementation of sterile insect technique (SIT) where economically feasible^24–26^. Current management strategies in Hawai’i include sanitation by removing old, dried or fallen fruit from orchards, chemical control by applying GF-120 Naturalyte Fruit Fly Bait (Spinosad) using a bait station approach, and mass trapping using yellow McPhail traps baited with torula yeast solution to attract female and male flies^7^. However, more recently biological control has become an important component of olive fly control in the United States^9,18^. A recent classical biological control effort in California involved the release of the braconid wasps *Psyttalia humilis* (Silverstri) and *Psyttalia loundsburryi* (Silvestri) from 2006 to 2013 following detection of the pest in that State in 1998. Over time, *P. lounsburryi* established in coastal regions, and given its success additional species have been proposed for release in California^27^.

Hawai’i has a long history of using braconid parasitoids wasps as biological control agents against tephritid fruit flies, reaching back to the early decades of the twentieth century^28^. The biology of these braconid agents has facilitated classical biological control programs in the archipelago^29,30^. In Hawai’i the earliest braconid introductions were to control the melon fly, *Zeugodacus cucurbitae* (Coquillett), which was detected in Hawai’i in 1895^31^. These efforts reached their peak in the years between 1947 and 1952, when 32 natural enemies were introduced against tephritid fruit flies, of which 26 were classified as braconid wasps at the time^29^. This remarkable rate of introductions together with advances in mass rearing and transportation of these species led to Hawai’i serving as a source for tephritid biological control programs around the world^31^. It also resulted in an unusually diverse assemblage of braconid wasps established in the Hawai’ian islands^32^, which provides the possibility of opportunistic biological control of *B. oleae* following its establishment.

The present study aimed to investigate the extent of opportunistic parasitoid activity against *B. oleae* in Hawai’i. We collected olive samples from 5 farm sites over two years on Hawai’i and Maui islands and reared out *B. oleae* to determine whether (a) parasitization was occurring naturally in the environment, (b) which species of parasitoid were most prevalent, (c) whether there were significant cultivar effects on *B. oleae* and parasitoid abundance^33^, and (d) if there was a seasonal pattern of parasitization that could inform future management decisions. We discuss the results in context of prospective augmentative biological control programs and the future of *B. oleae* management in Hawai’i and abroad.

## Results

### *Bactrocera oleae* abundance in olive samples

**Figure 1:**
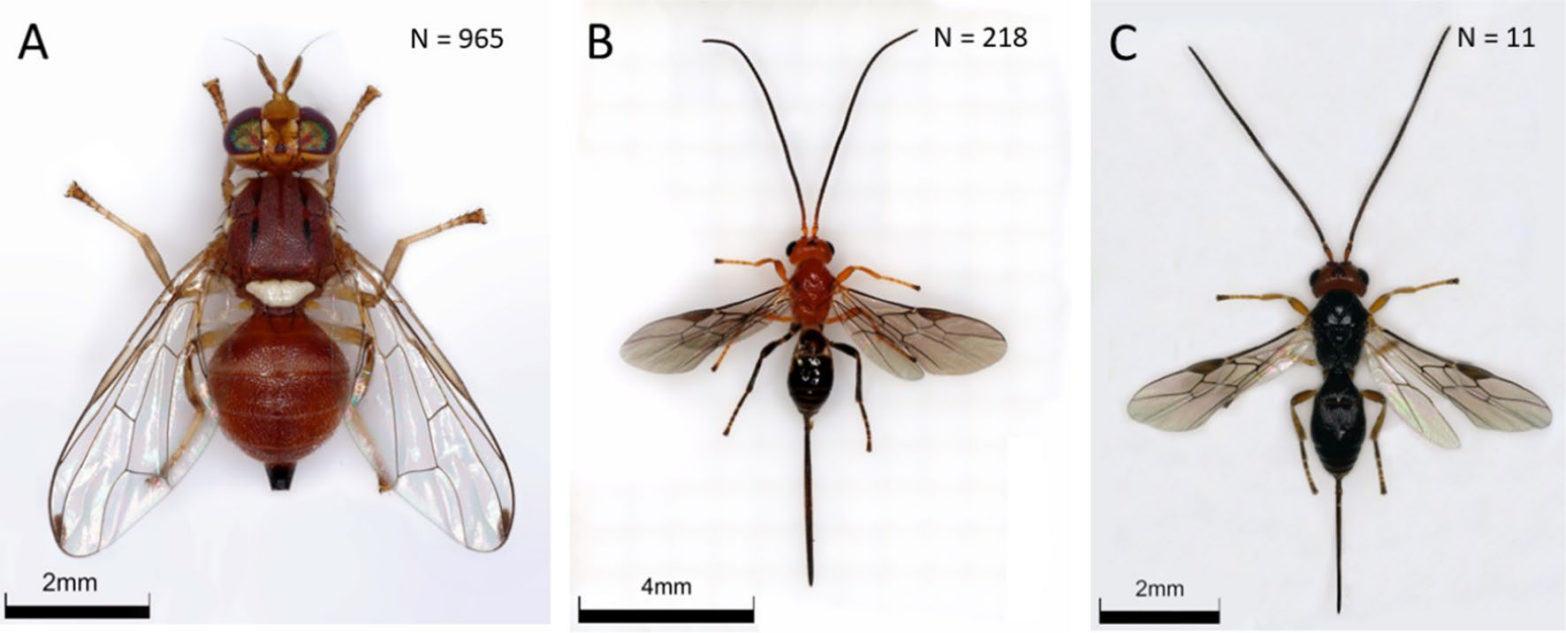
Images of *Bactrocera oleae* (**A**), *Diachasmimorpha tryoni* (**B**), and *Fopius arisanus* (**C**) reared from olive samples collected in Hawai’i during 2023. The total number of each species is shown as in the upper right corner of each image. Photographs courtesy of M. Weaver, ORISE fellow at USDA-ARS, Hilo, HI, 2023.

**Figure 2:**
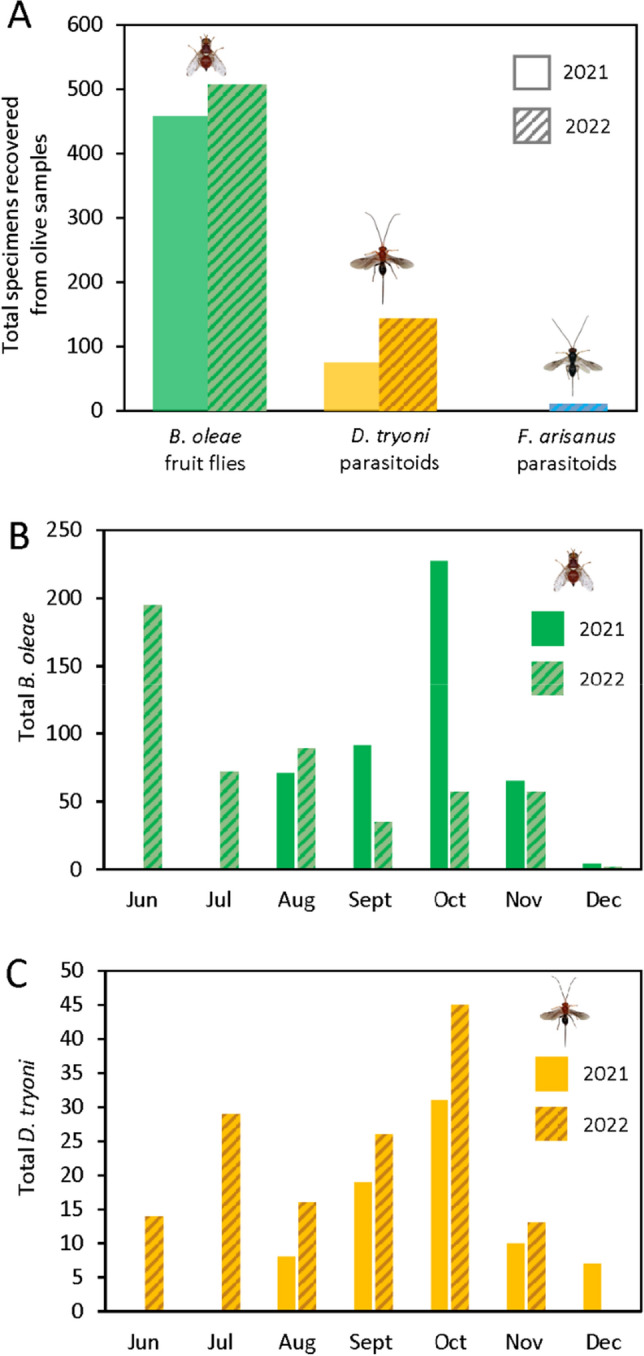
Comparative total emergence of the olive fruit fly *B. oleae* and two parasitoids *D. tryoni* and *F. arisanus* during 2021 and 2022 collections of olive fruit in Hawai’i (**A**). Total emergence for each month is shown for *B. oleae* (**B**), and *D. tryoni* (**C**) with solid bars indicating 2021 and patterned bars indicating 2022 data, respectively.

Olive fly was successfully recovered from all field collection sites included in the study. However, the greatest number came from Big Island samples. In total 311 olive flies emerged from McKanna samples, compared to 477 from Lalamilo, and 163 at Pohakuloa (Table 1, Fig. 1A). Only 8 total were obtained from Jaime’s farm samples, and 6 from Pueokea on Maui. Separated by year, in 2021 we reared a total of 458 *B. oleae* from 4089.1 g of olives (3649 olives), and 2022 we reared 507 *B. oleae* from 1773.3 g of olives (1894 olives) (Fig. 2A). Fewer olives were collected in 2022 due to a later season start date, based on fruit availability—2022 was a worse year for olives in Hawai’i in general with reduced yield on both Maui and Hawai’i. At McKanna, during 2021 we were able to collect for four months, while in 2022 it was reduced to only two months. Combining the data for the two years, poisson regression of *B. oleae* emergence by cultivar showed differences at Lalamilo (X^2^ = 24.63, df = 6,154, P < 0.001) and McKanna (X^2^ = 7.07, df = 1, 75, P = 0.008), but not Jaime’s farm (P = 0.81) or Pueokea (P = 0.91), but this may reflect larger sample sizes at the first two sites (Table S1). At Lalamilo, the mean *B. oleae* per gram from ‘Koroneiki’ (0.31 ± 0.07) was greater than reared from ‘Arbequina’ (0.15 ± 0.02) (Table 2). Meanwhile at McKanna the mean *B. oleae* per gram from ‘Koroneiki’ (0.08 ± 0.02) was less than from ‘Arbequina’ (0.34 ± 0.05) (Table 2).

**Table 1:** Differences in mean olive weight, parasitoid emergence, and olive fly emergence by site and olive variety during the 2021–2022 olive production period in Hawai’i.

Site location	Olive variety	N _trees_ =	Mean olive weight	*B. oleae*	*F. arisanus*	*D. tryoni*
Pohakuloa (950 m)	Unknown	15	0.99 ± 0.09	0.61^a^ ± 0.11 (163)^b^	0	0.14 ± 0.04 (34)
Lalamilo (800 m)	Arbequina	56	1.15 ± 0.05	0.15 ± 0.02 (153)	0.01 ± 0.00 (5)	0.08 ± 0.02 (89)
	Arbosana	27	0.90 ± 0.05	0.23 ± 0.05 (109)	0	0.02 ± 0.01 (9)
	Frantoio	16	1.16 ± 0.08	0.13 ± 0.03 (50)	0	0.03 ± 0.01 (11)
	Koroneiki	34	0.60 ± 0.04	0.31 ± 0.07 (109)	0.00 ± 0.00 (1)	0.04 ± 0.02 (13)
	Manzanilla de Sevilla	27	3.22 ± 0.13	0.03 ± 0.01 (81)	0.00 ± 0.00 (3)	0.02 ± 0.01 (32)
	Taggiasca	2	0.66 ± 0.07	0.07 ± 0.07 (2)	0	0.14 ± 0.14 (4)
	Unknown	5	1.35 ± 0.15	0.15 ± 0.03 (19)	0	0.09 ± 0.04 (10) a
McKanna (1450 m)	Arbequina	36	1.03 ± 0.03	0.34 ± 0.05 (242)	0	0.01 ± 0.01 (7) a
	Koroneiki	41	0.71 ± 0.03	0.08 ± 0.02 (69)	0	0.01 ± 0.00 (5)
Jaime (1060 m)	Arbequina	7	0.87 ± 0.08	0	0	0
	Koroneiki	15	0.44 ± 0.02	0.02 ± 0.02 (3)	0	0
	Leccino	8	1.57 ± 0.06	0	0	0
Pueokea (750 m)	Arbosana	14	0.77 ± 0.06	0.01 ± 0.01 (2)	0	0.01 ± 0.01 (2)
	Koroneiki	11	0.48 ± 0.03	0.05 ± 0.05 (6)	0	0.01 ± 0.01 (1)
	Moraiolo	15	2.19 ± 0.08	0	0	0
	Pendolino	2	1.33 ± 0.03	0.00 ± 0.00 (2)	0	0.02 ± 0.02 (1)

^a^Mean per gram of olives ± standard error.

^b^Total number collected for the whole trial in parentheses.

**Table 2:** Differences in *B. oleae* emergence from different olive cultivar samples at Lalamilo and McKanna sites where significant effects due to cultivar were indicated.

Site	Coefficient	Estimate	SE	Z-value	Pr > |z|	Tukey LSD
Lalamilo	(Intercept)	0.988^1^	0.083	11.894	** < 0.001**	
	Arbosana	0.408	0.127	3.217	**0.001**	b^2^
	Frantoio	0.111	0.171	0.650	0.516	ab
	Koroneiki	0.177	0.127	1.398	0.162	ab
	Manzanilla	− 0.029	0.167	− 1.769	0.077	a
	Taggiasca	− 0.988	0.712	− 1.387	0.165	ab
	Unknown	0.347	0.244	1.423	0.155	ab
	Arbequina (ref)					a
McKanna	(Intercept)	− 1.080	0.286	− 3.776	** < 0.001**	
	Koroneiki	− 1.503	0.636	− 2.362	**0.018**	a
	Arbequina (ref)					b

Significant values are in bold.

^1^Estimated means derived from poisson regression.

^2^α = 0.05.

### Parasitoid recovery

Parasitoids did emerge from olive samples and two species were identified (Table 1; Fig. 1B,C). The first parasitoid, *Diachasmimorpha tryoni* (Cameron) (Hymenoptera: Braconidae), was recovered from every site except Jaime’s farm on Maui (Fig. 1B; Table 1). In total, 218 *D. tryoni* were collected, with the greatest number (89) coming from ‘Arbequina’ cultivar samples at Lalamilo, χ^2^ = 601.86, DF = 6, 154; P < 0.001 (Table 3). During 2021 we recovered 75 total *D. tryoni* (Table S2), while in 2022 we recovered 143 (Fig. 2A; Table S3). The second parasitoid we recovered was *Fopius arisanus* (Sonan) (Hymenoptera: Braconidae) with a total of 9 recovered from olives collected at the Lalamilo site during 2022 (Fig. 1C; Table S3), and limited to ‘Arbequina’, ‘Koroneiki’, and ‘Manzanilla de Sevilla’ varieties. Two additional *F. arisanus* were reared out of ‘Moraiolo’ samples from Pueokea on Maui during June 2022 (Table S3). We did not find this parasitoid in our samples during 2021 (Table S2).

**Table 3:** Differences in *D. tryoni* emergence from different olive cultivar samples at Lalamilo during 2021–2022.

Coefficient	Estimate	SE	Z-value	Pr > |z|	Tukey LSD
(Intercept)	0.4997	0.1060	4.714	** < 0.001**	
Arbosana	− 1.5983	0.3498	− 4.569	** < 0.001**	a
Frantoio	− 1.8098	0.3196	− 2.534	**0.0113**	abc
Koroneiki	− 1.4611	0.2969	− 4.921	** < 0.001**	ab
Manzanilla	− 0.2120	0.2061	− 1.028	0.3038	c
Taggiasca	0.1935	0.5111	0.379	0.7050	bc
Unknown	0.1935	0.3335	0.580	0.5618	c
Arbequina (ref)					c

Significant values are in bold.

### Seasonality effects and fruit weight

The seasonality of olive fly and parasitoid emergence was different during 2021 and 2022, although collections were greatest October, coinciding with fruit availability (Fig. 2B,C). During 2021 at Lalamilo, peak olive fly numbers were recorded in August while *D. tryoni* numbers were greatest in October (Table S2). At McKanna, which is about 2000 ft higher in elevation, olive fly peaks occurred in October. During the following year in 2022, Lalamilo saw a peak in olive fly during June and then again during October (Table S3). Recovery of *D. tryoni* again peaked in October 2022 at this site. At McKanna, *B. oleae* was only collected during August but no parasitoids emerged from the samples. Instead, during 2022 samples from Pohakuloa showed peaks of *B. oleae* in June and *D. tryoni* in August. These patterns appear to correspond roughly to our subsampled data on total fruit weight at each site (Table S4), with more *B. oleae* present as fruit availability increased. However, infestation did not show patterns associated with mean fruit weight, indicating that this was not a good predictor of infestation risk or parasitoid abundance. The largest olives we sampled were the ‘Manzanilla de Sevilla’ at Lalamilo and the ‘Moraiolo’ at Peuokea farm, both of which were 2–3 × larger than ‘Arbequina’ olives but were associated with less than half the olive fly and parasitoid numbers on a per gram basis.

## Discussion

Our results show for the first time that previously introduced braconid parasitoids target the recent invader, *B. oleae*, in Hawai’i. Specifically, our data show that *D. tryoni* and in smaller numbers, *F. arisanus*, parasitize *B. oleae* in commercial olive orchards on the Big Island of Hawai’i and the island of Maui. This was not the result of intended augmentative releases, but rather a demonstration of opportunistic biocontrol^34,35^ wherein established populations of parasitoids, originally imported decades ago to combat other invasive pests in the state, appear to be targeting other newer invasive pests^31^.

The first parasitoid we detected was* D. tryoni*, a small opiine wasp that attacks late instar larvae of several species of Tephritidae; it was originally imported to Hawai’i from Australia in 1910 to combat the then-recently discovered Mediterranean fruit fly, *Ceratitis capitata* (Diptera: Tephritidae), which at the time was causing damage in Kona coffee fields, although this effect is now considered negligible as the fruit flies do not harm to the coffee beans themselves and merely feed and develop on the pulp surrounding the bean^36^. An interesting early report from 1914 described the limited ability of *D. tryoni* to access larvae deep within large fruit, thus it’s utility in small fruit orchards, such as coffee^36^. In addition to *C. capitata*, *D. tryoni* is also known to parasitize the Queensland fruit fly, *Bactrocera tryoni* (Froggart) in Australia^37,38^, the lantana gall fly (*Eutreta xanthochaeta* Aldrich)^39,40^, and very occasionally to two non-frugivorous tephritid flies*, Trupanea dubautiae* and *Ensina sonchi*, parasitism of which does not typically result in successful larval development^41^. In oriental fruit fly (*Bactrocera dorsalis* Hendel), *D. tryoni* only rarely parasitizes larvae, typically less than 1%^42^ and there are no reports of its attack on melon fly (*Z. cucurbitae*) or the Malaysian fruit fly (*Bactrocera latrifrons* Hendel), to our knowledge. Our study is the first to report natural parasitism of *B. oleae* by *D. tryoni* in Hawai’i or abroad^43^.

The second parasitoid we recovered from our samples was *F. arisanus*, a solitary, koinobiont opiine braconid targeting the egg-larval stages of tephritids. *Fopius arisanus* parasitoids have a larger host range than *Diachasmimorpha sp*. and often outcompete other braconid parasitoids^44^. One of the most successful braconid biological control agents against *Bactrocera* fruit flies in the Pacific and beyond; *F. arisanus* was brought to Hawai’i in 1948 to originally control *B. dorsalis*^31^. *Fopius arisanus* is also known to parasitize olive fruit fly^45^ and has been examined as a possible biological control agent for *B. oleae* management in California^46^ and internationally in Asia and Africa where it is a major crop pest^47^. Interestingly, *F. arisanus* has been cited in Israel as parasitizing olive fly despite its original introduction being to control *Ceratitis capitata*^48^; this is a very similar situation of opportunistic biological control as the one we report in the current study.

Our results provide helpful information in the effort to develop a successful management program for *B. oleae* in Hawai’i, but also suggests some questions. First, why were *D. tryoni* more abundant in our samples than *F. arisanus*? Second, is it likely that *F. arisanus* will supplant *D. tryoni* over time in olive fields of Hawai’i? It appears *F. arisanus* numbers are increasing year over year, and we can report that during late summer 2023 a single collection of olives from our Lalamilo site yielded the greatest numbers thus far—70 *F. arisanus*, 115 *D. tryoni*, and 190 *B. oleae* from approximately 2 kg of olives. This is in comparison to zero *F. arisanus* recovered from our 2021 samples, and only 11 total during 2022 for both Maui and Hawai’I islands, as we reported above. Although larval parasitoids such as *D. tryoni* and *D. longicaudata* have been used in successful biocontrol programs, they have generally been outcompeted and displaced by the egg parasitoid, *F. arisanus*^44,46,49,50^*.* This is in part due to overall lower parasitism rates for the *Diachasmimorpha* genus, which are generally below 10%^42^. In contrast, *Fopius* parasitoids are frequently associated with parasitism rates as high as 50% in the wild^51^. Further, the presence of *F. arisanus* is associated with high mortality rates (exceeding 80%) of *D. tryoni* eggs when laid in the same host due to inhibitory changes in the host hemolymph following initial parasitization^44,52^. Indeed, *F. arisanus* has been used to great success in the management of another economically significant *Bactrocera* species, *B. dorsalis* in Hawai’i and throughout the Pacific Basin since its original release in the 1940s^31,49,53,54^. However, *B.oleae* may not be as suitable a host for *F. arisanus* in Hawai’i given that olives are typically grown in Hawai’i at higher elevations. *Fopius arisanus* fecundity is maximized around 22 °C, which is slightly warmer than our olive sites’ mean daily temperature^55^.

Given the abundance of *D. tryoni* at the Hawai’i olive sites and association with similar climatic profiles, it could be a good option for release^56^, but more research is needed to compare the viability of both parasitoids for biocontrol under controlled conditions to confirm its suitability. Elevational effects on *D. tryoni* parasitism rates have been studied on Kauai in relation to the lantana gall fly *E. xanthochaeta* and were found in greatest numbers at upper elevations around 1000 m^57^. On Maui, a similar effect was noted where *D. tryoni* being used for management of *C. capitata* was greatest at an upper elevation Kula site on the western flanks of Haleakala at around 1200 m^58^. However, in our study, the site with the greatest *D. tryoni* parasitism was Lalamilo research station, which is situated at about 800 m (the other sites in our study ranged from 750 to 1450 m), suggesting elevation was not the only factor affecting parasitoid abundance we observed. Other abiotic climate factors may be at play, along with biotic effects such as general fruit abundance, factors affecting successful diapause, or interspecific competition^44^. We suggest that the presence of *D. tryoni* at these upper elevations may reflect existing overlap with its previously intended biological control target, Mediterranean fruit fly (*C. capitata*), which is most closely associated with coffee in Hawai’i. We noted an additional *C. capitata* host plant, *Solanum aculeatissimum* (Apple of Sodom) at the Pohakuloa site; and the Lalamilo site had an overgrowth of invasive lantana which is host to *E. xanthochaeta*. These resources may be recruiting and maintaining *D. tryoni* populations in these areas and help explain their relative abundance at these sites.

Our data suggest that augmentative release options may be warranted if an increase in parasitism were achieved following introduction in the field. However, assessments are required to confirm (a) that the local *F. arisanus* strain is a suitable parasitoid for *B. oleae* control and (b) that it performs well under local climatic conditions. We also acknowledge that logistics of rearing many influence the decision to rear one species of parasitoid over another for an augmentative release program. Further, there may be variation in competitive ability even within the islands and different parasitoids may perform better or worse at each location. For these reasons, while *F. arisanus* releases may be suitable at some Hawaiian olive sites, *D. tryoni* may be preferrable at others, particularly at higher elevations where *D. tryoni* is more competitive and *F. arisanus* less abundant in the environment. Future surveys to track naturally occurring interspecific competition in Hawai’i, along with an evaluation of rearing efficiency for both species in colony, will likely indicate the most suitable biological control option going forward.

## Methods

### Field sites

We studied three olive growing sites on the big island island of Hawai’i. The University of Hawai’i Lālāmilo Research Station in Waimea (hereafter referred to as “Lalamilo”) on Hawai’i Island (20°01′07″ N, 155°40′35″ W, elevation 2630 ft./800 m) is a site of ongoing olive cultivation research. Lalamilo has ten cultivars as well as ornamentals arranged in a randomized block formation within a 1.6 hectare (4 acre) plot on the research farm. McKanna farm at Waikii ranch (hereafter referred to as “McKanna”, Waimea, Hawai’i (19°51′11″ N, 155°38′55″ W, elevation 4750 ft/1450 m) is a commercial, high-density orchard on 0.2 hectares (0.5 acres) with approximately 200 productive trees planted in 2014, comprising two cultivars, Koroneiki (40) and Arbequina (159) and arranged in three parallel rows. The U.S. Army Garrison—Pōhakuloa Training Area (19°50′15.5″ N, 155°42′44.7″ W, elevation 3150 ft./950 m) (hereafter referred to as “Pohakuloa”) contains a now wild population of thousands of olive trees (cultivars unknown) with their epicenter covering an area of around 140 hectares (350 acres). All of the trees reportedly stem from an original planting of seven trees presumably from the late 19th to early twentieth century, and an additional 40 trees planted in the 1940s.

The two additional olive growing sites were located on the neighboring island of Maui at oil producing farms in the Kula region of the island. The upper site, Jamie’s Farm (hereafter referred to as “Jaime” (20°44′09″ N, 156°19′28″ W, elev. 3500 ft/1060 m), covers 8 hectares (20 acres) with approximately 400 trees in production and 12 cultivars. This orchard has been in production for 7 years. The lower site, Lei’s Farm at Pueokea (hereafter referred to as “Pueokea” (20°46′23″ N, 156°19′43″ W, elevation 2434 ft/750 m) is approximately 7.3 hectares (3.3 acres) with roughly 1200 trees and 10 cultivars. This orchard has been in production for 6 years.

### Sample collection

To investigate parasitoids of olive fruit fly in Hawai’i, potentially infested olives were harvested and kept in a controlled environment to allow emergence of target species. Every 1–2 weeks, 20 olives were collected from designated trees at 1–2 m height as available in the canopy. Collection occurred from the first visible instance of infestation until either harvest or the last olives naturally dropped. While samples were collected from Lalamilo and McKanna during 2021 and 2022, samples from Pohakuloa were only collected during the 2022 season. Data were collected from Jaime’s farm during August and October of 2021, and June–August of 2022. Pueokea was added during the 2022 season only.

Trees at Lalamilo were selected based on those that had enough fruit to support collection throughout the season. For the first season, the twelve trees selected represented five cultivars: Arbequina (4), Arbosana (2), Frantoio (2), Leccino (1), and Manzanillo de Sevilla (2). Some fruit collection trees coincided with those that bore olive fly multilure traps. The second season was impacted by an irregular fruiting season as well as the biennial fruiting nature of some cultivars, hence the difference in producing varieties. At the McKanna farm site, picking was randomized within the two main cultivars (Koroneiki and Arbequina). Trees that had fruit picked one week were flagged to be avoided for the remainder of the season so that no tree was picked from twice. The second season was impacted by heavy pruning so only large batch sampling of a handful of trees was possible. Only five producing trees were used at Pohakuloa given irregular and dangerous terrain.

### Insect rearing

Once collected, olives were brought back to the lab in Hilo (USDA-ARS), weighed (Sartorius, Entris BCE), and placed in 16 oz. deli cups (473 mL) with mesh fitted lids. Cups were held in growth chambers (temp: 25.5 °C, RH: 65%, 12:12 light cycle) for two months with flies and parasitoids removed periodically as they emerged. Parasitoids were placed in plastic scintillation vials with 95% ethyl alcohol and kept in at – 40 °C. Morphological identification of parasitoids was possible through both a dichotomous key (Wharton, RA and Yoder, MJ. Parasitoids of Fruit-Infesting Tephritidae, http://paroffit.org) as well as comparison to an existing pinned collection.

### Statistical analysis

All analysis was conducted using R statistical software V 4.2.0 (R Foundation for Statistical Computing, Vienna, Austria.) We compared insect emergence from olive samples among cultivars using a generalized linear model with Poisson distribution due to negative data skew^59^. Since some varieties possessed large olives than others, we analyzed our data on a per gram of olive basis. Goodness of model fit was estimated using analysis of deviance using the package “car”^60^. Posthoc tukey’s LSD comparisons of mean emergence was compared using the package “emmeans”^61^.

### Ethics

According to journal policies involving experimental research and field studies on plants (either cultivated or wild), we declare that our research complies with relevant institutional, national, and international guidelines and legislation. All plant material was collected with the permission of the farms on which we worked, and no indigenous plants or animals were harmed in the process.

### Supplementary Information

Supplementary Information 1.

Supplementary Tables.

## Data Availability

All data generated or analysed during this study are included in this published article [and its supplementary information files].
