# Supplementary material for: Previously introduced braconid parasitoids target recent olive fruit fly (Bactrocera oleae) invaders in Hawai’i
Source: Sci Rep. 2023 Dec 18;13:22559. doi: 10.1038/s41598-023-49999-x (PMC10728124; doi:10.1038/s41598-023-49999-x)
Supplement: Supplementary file 2 — Supplementary Tables. [file 41598_2023_49999_MOESM2_ESM.docx]

**Supplemental**

**Table S1.** Statistical differences (bold) between cultivars at each site in mean *B. Oleae, F. arisanus,* and *D. tryoni* emergence on per gram of olive basis.

| **Collection site** | **Species collected** | **X2** | **DF** | **P** |
| --- | --- | --- | --- | --- |
| Lalamilo | *B. oleae* | 24.63 ^a^ | 6, 154 | **<0.001** |
|  | *F. arisanus* | 8.59 | 6, 154 | 0.198 |
|  | *D. tyroni* | 60.86 | 6, 154 | **<0.001** |
| McKanna | *B. oleae* | 7.07 | 1, 75 | **0.008** |
|  | *F. arisanus* | --- ^b^ | --- | --- |
|  | *D. tyroni* | 0.01 | 1, 75 | 0.923 |
| Jaime | *B. oleae* | 0.43 | 2, 26 | 0.81 |
|  | *F. arisanus* | --- | --- | --- |
|  | *D. tyroni* | --- | --- | --- |
| Pueokea | *B. oleae* | 1.00 | 4, 38 | 0.909 |
|  | *F. arisanus* | 0.10 | 4, 38 | 0.999 |
|  | *D. tyroni* | 0.34 | 4, 38 | 0.987 |

^a^ Goodness of fit for poisson regression by species (test = “Chisq”).

^b^ Analysis removed due to zero count data for that species at the specified location.

**Table S2.** Total number of *B. oleae, F. arisanus*, and *D. tryoni* collected each month during the olive fruiting period of 2021.

| **Collection site** | **Species collected** | **Jul** | **Aug** | **Sep** | **Oct** | **Nov** | **Dec** |
| --- | --- | --- | --- | --- | --- | --- | --- |
| Lalamilo | *B. oleae* | . ^a^ | 66 ^b^ | 49 | 39 | 21 | 4 |
|  | *F. arisanus* | . | 0 | 0 | 0 | 0 | 0 |
|  | *D. tyroni* | . | 8 | 19 | 25 | 5 | 6 |
| McKanna | *B. oleae* | . | . | 42 | 188 | 44 | 0 |
|  | *F. arisanus* | . | . | 0 | 0 | 0 | 0 |
|  | *D. tyroni* | . | . | 0 | 6 | 5 | 1 |
| Jaime | *B. oleae* | . | 5 | . | 0 | . | . |
|  | *F. arisanus* | . | 0 | . | 0 | . | . |
|  | *D. tyroni* | . | 0 | . | 0 | . | . |
| Pueokea | *B. oleae* | 10 | 7 | . | . | . | . |
|  | *F. arisanus* | 0 | 0 | . | . | . | . |
|  | *D. tyroni* | 7 | 0 | . | . | . | . |

^a^ (.) Indicates no fruit available to collect during this period.

^b^ Total number of collected insects (by species) during that particular month for all trees at each site.**Table S3.** Total number of *B. oleae, F. arisanus*, and *D. tryoni* collected each month during the olive fruiting period of 2022.

| **Collection site** | **Species collected** | **Jun** | **Jul** | **Aug** | **Sep** | **Oct** | **Nov** | **Dec** |
| --- | --- | --- | --- | --- | --- | --- | --- | --- |
| Pohakuloa | *B. oleae* | 88 ^a^ | 32 | 43 | . ^b^ | . | . | . |
|  | *F. arisanus* | 0 | 0 | 0 | . | . | . | . |
|  | *D. tyroni* | 3 | 15 | 16 | . | . | . | . |
| Lalamilo | *B. oleae* | 101 | 40 | 6 | 35 | 57 | 57 | 2 |
|  | *F. arisanus* | 3 | 0 | 1 | 1 | 1 | 3 | 0 |
|  | *D. tyroni* | 7 | 14 | 0 | 26 | 45 | 13 | 0 |
| McKanna | *B. oleae* | . | . | 37 | 0 | . | . | . |
|  | *F. arisanus* | . | . | 0 | 0 | . | . | . |
|  | *D. tyroni* | . | . | 0 | 0 | . | . | . |
| Jaime | *B. oleae* | 0 | 0 | 3 | . | . | . | . |
|  | *F. arisanus* | 0 | 0 | 0 | . | . | . | . |
|  | *D. tyroni* | 0 | 0 | 0 | . | . | . | . |
| Pueokea | *B. oleae* | 6 | 0 | 0 | . | . | . | . |
|  | *F. arisanus* | 2 | 0 | 0 | . | . | . | . |
|  | *D. tyroni* | 4 | 0 | 0 | . | . | . | . |

^a^ Total number of collected insects (by species) during that month for all trees at each site.

^b^ (.) Indicates no fruit available to collect dur

**Table S4.** Total (and mean) olive weight at each olive collection site by month during 2021 and 2022.

| Year | Site | **Jun** | **Jul** | **Aug** | **Sep** | **Oct** | **Nov** | **Dec** |
| --- | --- | --- | --- | --- | --- | --- | --- | --- |
| 2021 | Jamie | NA | . | 128.73 (1.41) | . | 112.34 (0.64) | . | . |
|  | Lalamilo | NA | . | 658.78 (1.37) | 757.52 (1.58) | 602.53 (1.31) | 568.25 (1.42) | 212.86 (2.13) |
|  | McKanna | NA | . | . | 100.25 (0.63) | 522.35 (0.87) | 381.79 (0.95) | 284.77 (0.95) |
|  | Pueokea | NA | NA | NA | . | . | . | . |
| 2022 | Jamie | 162.23 (0.81) | 162.56 (0.82) | 140.3 (0.91) | . | . | . | . |
|  | Pohakuloa | 110.26 (1.10) | 80.66 (0.81) | 74.87 (1.06) | . | . | . | . |
|  | Lalamilo | 212.11 (1.06) | 194.8 (1.22) | 39.9 (2.00) | 225.52 (1.04) | 391.28 (1.22) | 269.49 (1.04) | 38.43 (0.44) |
|  | McKanna | . | . | 60.38 (0.46) | 75.6 (0.45) | . | . | . |
|  | Pueokea | 388.42 (1.08) | 370.83 (1.17) | 225.25 (1.79) | . | . | . | . |
